# A Prospective Cross-Sectional Study on the Comparison of Ultrasound Assessment vs. Palpation in Chronic Lymphocytic Leukemia Patients in the Era of Targeted Therapy

**DOI:** 10.3390/jcm11113206

**Published:** 2022-06-04

**Authors:** Edoardo Benedetti, Fabrizio Mavilia, Claudia Baratè, Emilia Bramanti, Riccardo Morganti, Giulia Cervetti, Enrico Capochiani, Benedetto Bruno, Matteo Pelosini, Salvatore Massimo Stella, Sara Galimberti

**Affiliations:** 1 Azienda Ospedaliero Universitaria Pisana, Department of Clinical and Experimental Medicine, UO Hematology, University of Pisa, 56126 Pisa, Italy; fabriziomavilia@gmail.com (F.M.); claudia.barate@gmail.com (C.B.); juliacervetti@alice.it (G.C.); mpelo78@hotmail.com (M.P.); sara.galimberti@unipi.it (S.G.); 2 Italian School of Basic and Emergency Ultrasound (SIUMB), 56100 Pisa, Italy; stellasalvatoremassimo@gmail.com; 3 Institute of Chemistry of Organometallic Compounds (ICCOM), Italian National Research Council (CNR), Via G Moruzzi 1, 56124 Pisa, Italy; bramanti@pi.iccom.cnr.it; 4 Section of Statistics, Azienda Ospedaliero Universitaria Pisana, 56126 Pisa, Italy; r.morganti@ao-pisa.toscana.it; 5 Azienda USL Toscana Nord Ovest, Hematology, 57124 Livorno, Italy; enrico.capochiani@gmail.com; 6 Department of Molecular Biotechnology and Health Sciences, University of Turin, 10126 Turin, Italy; benedetto.bruno@unito.it

**Keywords:** lymph node, ultrasound sonosgraphy, spleen, chronic lymphocytic leukemia

## Abstract

Background. In IWCLL guidelines, progressive splenomegaly and lymphadenopathy are signs of active disease. In this study, we have tested the hypotheses if US could be a reliable tool for both superficial lymphnodes (SupLNs) and splenic assessment in chronic lymphocytic leukemia (CLL) patients. Methods. We enrolled N = 75 patients. SupLN and the spleen were assessed by two independent physicians (M1 and M2) by palpation and by a third physician (M3) with ultrasound sonography (US) using two different sonographers (US1 and US2). The results of M1 vs. M2 assessment, US1 vs. US2, palpation vs. US were compared. The echostructure of N = 1037 SupLN and of the spleen was also investigated. Results. The dimensions of SupLNs assessed by MD1 vs. MD2 were statistically discordant. Splenic size was concordant. There was concordance between US1 and US2 SupLN and splenic assessment. US found a higher number of pathological SupLN (Cohen’s Kappa < 0.1) than palpation, which misses remarkable-sized SupLNs. LN echostructure and splenic involvement patterns were described. Conclusions. US is a reliable, radiation-free tool useful in clinical practice to assess SupLN and splenic involvement in CLL.

## 1. Introduction

The 2018 International Workshop on Chronic Lymphocytic Leukemia (IWCLL) guidelines [1], outside the context of clinical trials, suggested ultrasonography (US) as a possible imaging technique to evaluate visceral involvement, and palpation to evaluate superficial lymph nodes (SupLNs). CT scan is not recommended outside the context of clinical trials, because is considered potentially harmful [1]. Ultimate ultrasonography machines are equipped with high-resolution linear probes, and US features of normal and diseased SupLNs have been published [2,3,4]. Criteria to define active CLL disease have been reported [1,5], and include progressive splenomegaly and progressive lymphadenopathy. In the era of chemotherapy-free CLL targeted treatments (such as Bruton’s tyrosine kinase, BTK, and B-cell lymphoma 2 protein, BCL2, inhibitors), patients assessment is important to start a novel agent at the correct timing, in order to obtain a deep response [6,7,8] such as complete remission (CR) of disease [9], a better progression-free survival (PFS) [9,10], and possibly to obtain a state of minimal residual disease negativity [9].

The aim of this prospective study has been to verify if, in N = 75 patients affected by CLL, US could assess the disease in SupLNs and spleen compared to palpation. First, we addressed the degree of concordance between two different physicians in the assessment of both superficial LN and of the spleen using palpation. Second, we addressed the degree of concordance between two different US sonographers in the assessment of SupLN and splenic dimensions. Third, we investigated the concordance or discordance between palpation vs. US for SupLN (number and dimensions of pathological CLL SupLN and splenic dimensions). Furthermore, we analyzed the echo structure of CLL SupLN and the splenic pattern of involvement.

## 2. Materials and Methods

### 2.1. Location, Patients, and Study Design

This study was conducted at the Hematology Unit of the University of Pisa, Italy, and all patients signed a written consent form. This study was performed in line with the principles of the Declaration of Helsinki (Ethical Committee approval Nr. 12174, 4 March 2020).

We enrolled N = 75 consecutive patients affected by CLL. Patients’ characteristics were as follows: gender N = 22 females and N = 53 males, median age = 69 years old (range 43–86 years), and median BMI 24.2 (range 17.3–33.6). Patients enrolled in the study were either in follow up without any previous treatment (N = 7), or in follow up post chemo-immunotherapy (N = 18; N = 7/18 post Benadamustine-Rituximab, N = 9/18 post FRC, N = 2/18 post chlorambucil-Rituximab), or on treatment (Ibrutinib N = 16; Venetoclax alone N = 11; Venetoclax-Rituximab N = 13; chlorambucil-Rituximab N = 10). Each patient was assessed, during the same outpatient clinic, by two independent physicians (MD1 and MD2), blinded to each other, with palpation of SupLN and spleen as previously reported [1,5]. MD1 and MD2 were hematologists with clinical expertise in lymphoproliferative disorders. A third physician (MD3), blinded to MD1 and MD2, assessed with US both splenic dimensions (cranio-caudal diameter and cross-sectional area) [4,11,12] and SupLNs [2,3,4,11]. The US assessment was performed for each patient by MD3, using two different US sonographers: Esaote Class-C-Advance (US1) and a General Electric Logiq e10s (US2). Briefly, 2–5 MHz bandwidth (GE) and 3.5–5 MHz (Esaote) convex probe were used for abdominal scanning, and a linear matrix array probe bandwidth of 4–16 MHz (GE) and a 7.5 MHz linear probe (Esaote) were used for SupLN assessment. The physician MD3 who performed the US examinations was a hematologist member and teacher at the Italian School for basic and emergency ultrasound (SIUMB) of the University of Pisa with more than 20 years’ experience in lymphoproliferative disorders and US [11,13,14,15,16,17,18]. Parameters assessed by palpation of SupLNs were: presence or absence of LNs in the superficial palpable bilateral regions (inguinal, axillary, cervical, and supraclavicular) [1,19]; if SupLNs were palpable, they were classified as pathological if >1 cm according to what previously reported [1,19]. Splenic dimension was assessed by palpation by MD1 and MD2 for each patient, as previously described [1,19] and if splenomegaly was detected, MD1 and MD2 reported the distance in cm below the costal margin [1,19]. 

**Figure 1:**
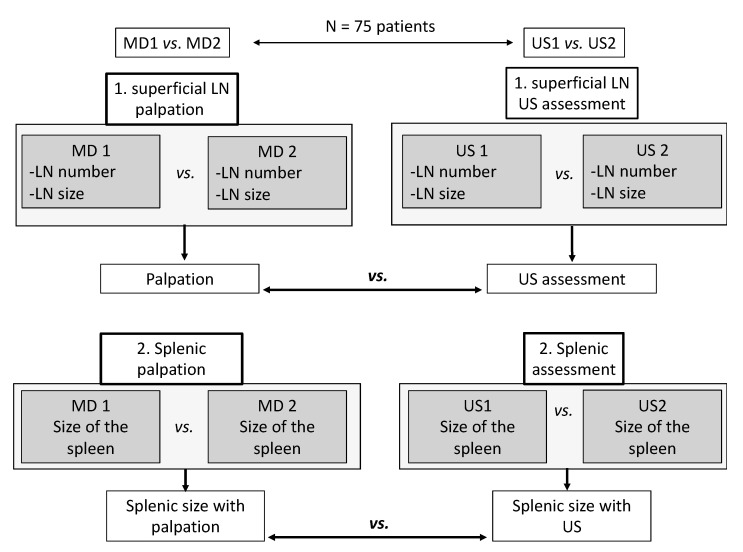
Schematics of the study design.

US assessment (with US1 and US2) involved the bilateral inguinal, axillary, cervical, supraclavicular, and subclavicular regions. For each superficial anatomical region, the parameters assessed were the number of pathological LN, their dimensions (in mm), the echo structure, and vascular pattern [20,21,22,23]. Using US, SupLNs were defined as either pathological or reactive or liposclerotic as previously described [4,11,18,23]. The echo structure of N = 1037 total CLL SupLNs found in N = 75 parents enrolled in the study was also analyzed. The spleen was assessed with US1 and US2 for each patient describing splenic longitudinal diameter, the cross-sectional area, as previously reported [11,18,23], and the pattern of CLL involvement. In this study, we choose to assess the splenic dimensions rather than the liver, with palpation (MD1 and MD2) and with US (US1 and US2) because only progressive splenomegaly is listed among the criteria to define active disease in the 2018 IWCLL and 2020 ESMO guidelines [1,19,23]. In our work, palpation was considered the standard approach in general practice (IWCLL 2018 guidelines), and US was considered the investigational arm. Figure 1 shows a schematic of our study design.

### 2.2. Statistics

Categorical data were described by absolute and relative frequency, continuous data by mean, and standard deviation. To analyze the agreement between two physicians related to palpation Cohen’s kappa test, *t*-test for independent data and Pearson’s correlation analysis were performed, while to analyze the agreement between physicians related to ultrasound, Bland–Altman plot was applied. The comparison between palpation and ultrasound categorical assessment was obtained by Cohen’s kappa test. Significance was fixed at 0.05 and all analyzes were carried out with SPSS v.27. 

## 3. Results

### 3.1. Comparison of Superficial LN and Splenic Palpation between MD1 and MD2

Table 1 reports the statistical results of the comparison of the total number of pathological SupLNs found bilaterally with palpation in each superficial anatomical region by MD1 and MD2. Statistics show concordance between MD1 and MD2. SupLNs ≥ 10 mm were considered pathological in all superficial anatomical regions [1,19].

Table 2 shows the statistical results of the comparison between MD1 and MD2 related to the assessment of the dimensions of pathological cervical, axillary, and laterocervical SupLNs using palpation. In the inguinal region, the comparison of the means of LN size assessed by MD1 and MD2 was statistically discordant, although Pearson’s r showed a positive correlation. In the laterocervical region, the comparison of the means of LN size assessed by MD1 and MD2 was concordant, and Pearson’s r showed a positive correlation. In the axillary superficial anatomical region, the means of LN size were statistically discordant, and no correlation was observed.

Nevertheless, we found concordance between MD1 and MD2 in the description of splenic dimensions, as MD1 and MD2 revealed splenomegaly in the same number of patients (N = 10 out of 75 patients assessed), and there was concordance in the description of the splenic size (cm below the costal margin) [1,5,19] (Cohen’s kappa = 0.874). 

### 3.2. Comparison of Superficial LN and Spleen Assessment Using Two Different US Sonographers

The number of pathological superficial LN in all superficial anatomical regions assessed using the two US sonographers (US1 vs. US2) was not statistically different (Cohen’K = 0.760).

**Figure 2:**
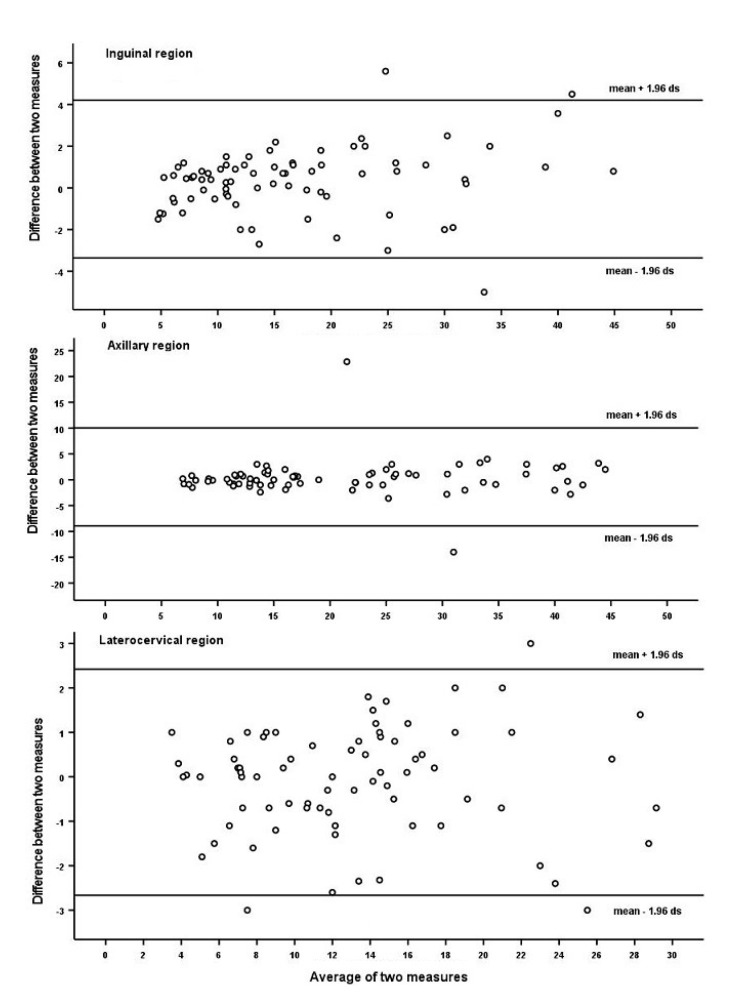
Bland–Altman plots related to the comparison of the dimensions of bilateral SupLNs in the inguinal, axillary, and laterocervical regions (US1 vs. US2). US1 = Esaote ultrasonographer; US2 = GE ultrasonographer; dimensions in mm.

Figure 2 and Table 3 report the results of the assessment of the dimensions of SupLNs and spleen using two different US sonographers. Figure 2 shows the Bland–Altman plots related to the comparison of the dimensions of bilateral SupLNs in the inguinal, axillary, and later cervical regions, showing non-statistical differences between the results obtained by the two sonographers (US1 vs. US2). Table 3 shows the comparison of the dimensions of bilateral SupLNs in the sub- and supraclavicular regions and of the spleen (US1 vs. US2).

The dimensions of SupLNs in all superficial anatomical regions and spleen obtained with US1 and US2 were not statistically different. The last two columns of Table 3 show that the concordance in reporting the dimensions of the LNs was independent of LN size.

### 3.3. Comparison between Palpation and US

#### 3.3.1. Detection of Pathological SupLN and Splenomegaly by US over Palpation

Table 4 shows the results of the comparison between US vs. palpation for both SupLNs and spleen. For each superficial anatomical region, we compared the number of pathological LN detected either by palpation or by US. Due to the non-statistical difference between the results obtained by the two US machines (US1 and US2), we compared the results of US1 with the results of palpation. SupLN was defined as pathological if ≥10 mm with palpation [1,19], and for each SupLN region, US results were compared with the results of the physician (either MD1 or MD2) who described the biggest dimensions of the pathological LNs, and consequently the highest number of pathological LN. Despite the comparison criteria adopted would favor the assessment by palpation, we found statistically different results between palpation and US assessment, as palpation missed both a significant number of pathological LN for each superficial region and splenomegalic patients (Table 4).

Overall, US vs. palpation detected N = 119 vs. N = 19 and N = 121 vs. N = 24 pathological LN in right and left inguinal regions, respectively. US vs. palpation detected N = 169 vs. N = 8 and N = 173 vs. N = 5 pathological LN in right and left axillary regions, respectively. US vs. palpation detected N = 165 vs. N = 17, and N = 180 vs. N = 10 pathological LN in right and left laterocervical regions, respectively.

In supra- and subclavicular regions, overall US found N = 37 and N = 43 right and left subclavicular pathological LN, respectively, and N = 13 and N = 9 right and left supraclavicular pathological LN, which were not detected with palpation. 

Splenomegaly was found with US vs. palpation and in N = 61/75, and N = 10/75 patients, respectively. Thus, palpation missed the detection of N = 39 patients in which the spleen resulted enlarged to US inspection.

#### 3.3.2. Dimensional Assessment of Pathological SupLNs and Splenomegaly Detected by US Missed by Palpation

Table 5 shows the dimensions of the pathological SupLNs analyzed for each superficial anatomical region that were not detected by palpation (median and range in mm) and of the spleen (median and range of the longitudinal diameter in cm, and area in cm^2^). The data reported for the spleen are related to the organ not considered enlarged with palpation that instead was found enlarged by US scanning.

The results reported in Table 5 clearly show that palpation missed LNs with a diameter of up to 54 mm in the axilla, up to 45 mm in the inguinal region, and up to 34.5 mm in the laterocervical region. Moreover, US found 30 mm diameter LNs in the subclavicular region, which are inaccessible with palpation.

#### 3.3.3. Impact of BMI and Age on Discordance between Palpation and US

We tested if the discordance found between palpation and US could depend on patient age or BMI. Age or BMI does not have a statistically significant impact on splenic and SupLN assessment (for age *p* = 0.448 and *p* = 0.322, respectively; for BMI *p* = 0.642, and *p* = 0.607, respectively).

### 3.4. Ultrasound Echostructure of CLL SupLNs and Splenic Involvement Pattern

#### 3.4.1. Ultrasound Echostructure of CLL SupLNs

**Figure 3:**
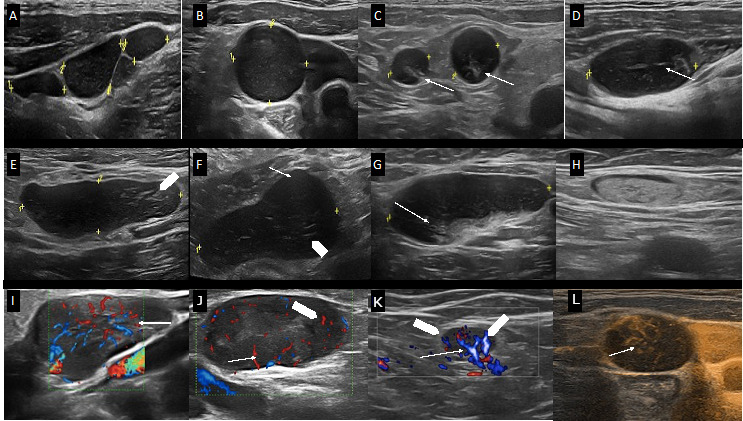
(**A**) N = 4 later cervical CLL SupLNs (14.9 mm, 16.4 mm, 11.7 mm, 7.9 mm). The SupLNs appear hypoechoic, without a US visible hilum, L/S < 2. The cortex appears thickened and inhomogeneous with reticulation. The SupLN present “chain”-shaped, contiguous, sharp borders. (**B**) Axillary CLL LN (13.6 mm). The LN appears hypoechoic, without US visible hilum, L/S ratio <2, with thickened, reticulated cortex, and sharp, regular borders. (**C**) Two inguinal CLL LNs (9.0 and 6.5 mm). The LNs appear hypoechoic, L/S ratio <2, with sharp borders and US-detectable hilum, although displaced and truncated (white arrowhead). The cortex is thickened, with reticulation. (**D**) Example of axillary CLL LN: L/S ratio >2, with visible, narrowed hilum, compressed by the thickened cortex. The cortex appears inhomogeneous (the posterior part more thickened than the anterior part). The reticulation of the cortex is prominent. (**E**) Axillary CLL LN, L/S ratio >2, with nonvisible hilum, sharp borders, and lobular shape. The cortex has an inhomogeneous thickening and reticulation (white arrowhead). (**F**) Axillary hypoechoic CLL LN, L/S ratio >2, sharp borders, without US visible hilum, with thickened, reticulated cortex (white arrow); the cortex has a polar cortical thickening (white arrowhead). (**G**) Axillary CLL LN (43.3 mm), L/S ratio >2, inhomogeneous, reticulated, and thickened cortex; the LN hilum is displaced and truncated (white arrowhead). (**H**) Example of axillary CLL LN in complete remission after treatment (liposclerotic). (**I**) Example of the color-doppler hilar pattern of a representative CLL axillary LN in which vessels are preserved, with regular branches reaching the peripheral part of the LN. (**J**) Example of chaotic, mixed color-doppler pattern of a CLL LN: sub-capsular vessels (white arrow) and intra-nodular vessels are displaced (white arrowhead). (**K**) Example of a hilar color-doppler pattern vascularization (white arrow) and subcapsular vascularization (white arrowheads) in the same CLL LN. (**L**) US-BMODE of CLL LN and vascular network using hybrid B-flow capture for enhanced microvascular details: white arrow shows the principal hilar vessel and white arrowhead shows vessels displaced irregularly, with subcapsular vessels, making the vessels network pattern overall chaotic. In panels “+” signs indicate the position of the calipers to measure the LN dimensions; “1” and, where present, “2” indicate different LNs.

We analyzed the US features of N = 1037 total CLL SupLNs detected in the N = 75 patients enrolled in the study (Figure 3) [11,24,25,26].

**Figure 4:**
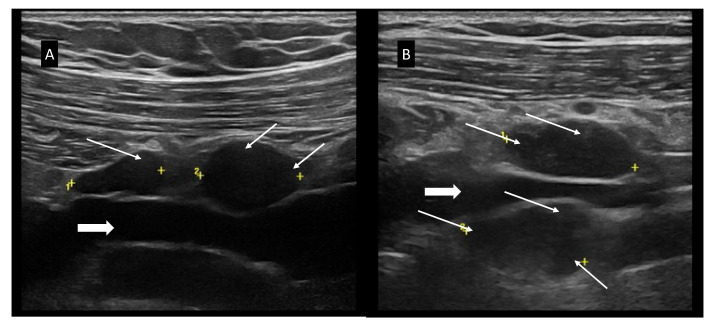
(**A**) Right SC SupLNs of 13.6 mm and 14.9 mm; (**B**) SC left SupLNs of 21 mm and 19.6 mm. The LNs are hypoechoic, without a visible hilum, with thickened, reticulated cortex. White arrows show a focal thickening of the cortex. Arrowheads indicate the subclavian vein. The SC SupLNs were not assessable with palpation. In panels “+” signs indicate the position of the calipers to measure the LN dimensions; “1” and, where present, “2” indicate different LNs.

**Figure 5:**
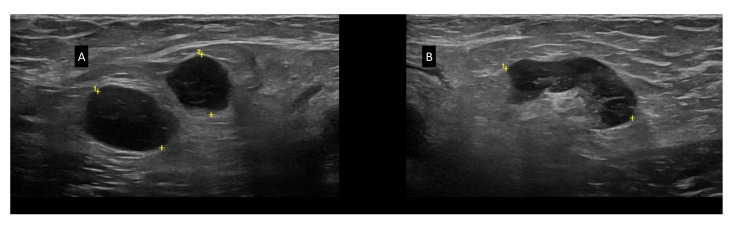
BMODE-US features contextually present in the same patient affected by CLL. (**A**) Left Ing LNs. (**B**) Right Ax LNs. The LNs of panel (**A**) coexist with the LNs found in the left inguinal region described in Figure 3 panels (C,D). In panels “+” signs indicate the position of the calipers to measure the LN dimensions; “1” and, where present, “2” indicate different LNs.

Figure 3 shows US characteristics of BMODE, color doppler, and hybrid B-flow capture for enhanced microvascular details assessment. In BMODE analysis, CLL LNs may have different patterns: (i) round shape (L/S < 2), without US-detectable hilum, but with a thickened, reticulated cortex; (ii) round shape (L/S < 2), with US-detectable hilum, although displaced, or truncated, or compressed (narrowed hilum), with thickened and reticulated cortex; (iii) oval shape (L/S > 2), without hilum, with irregularly and eccentric [22], thickened, reticulated cortex that determines the lobular profile of the LN, or detectable hilum that appears compressed (narrowing), displaced or truncated due to the eccentric, thickened, reticulated cortex (Figure 3E) [27]. In all LNs scanned, we found well-defined borders and we never found US signs of extracapsular spread as in metastatic LNs [21,22]. Figure 3 panels I, L, M, and N show the various color Doppler patterns classified as: (i) hilar, (ii) mixed subcapsular and hilar, and (iii) chaotic [20]. Moreover, we found that in CLL patients, SupLNs (Figure 4) may have different US aspects in different or even in the same anatomical region (Figure 5).

#### 3.4.2. Ultrasound Patterns of Splenic CLL Involvement

**Figure 6:**
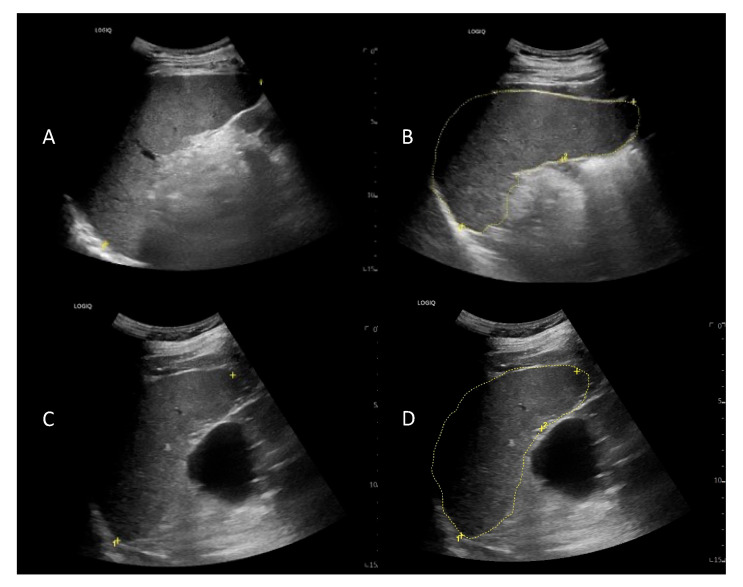
US scan of spleen in CLL patient. In (**A**,**B**) the echostructure of the spleen is finely inhomogeneous. Both longitudinal diameter (**A**) and cross-sectional area (**B**) are enlarged (15 cm and 77.7 cm^2^, respectively). In panel (**C**,**D**), the echostructure of the spleen is finely inhomogeneous. Longitudinal diameter is within normal range (11.9 cm, panel (**C**)), but cross-sectional area is increased, showing that the spleen is overall enlarged (57.8 cm^2^, panel (**D**)). In panels “+” signs indicate the position of the calipers to measure the spleen dimensions; “1” indicates the splenic longitudinal diameter, and “2” indicates the initial point to initiate to calculate the cross-sectional area of the spleen.

US detected N = 39/75 splenomegaly cases. In N = 27/39 patients, splenomegaly resulted from both increased longitudinal diameter (median length of 12.7 cm, range 12.6–28 cm) and cross-sectional area (median dimensions of 61 cm^2^, range 47–150 cm^2^). In N = 12/39 patients the longitudinal diameter of the spleen was within the normal range, but the cross-sectional area was increased with median dimensions of 51.5 cm^2^ (range 50–97 cm^2^) (Figure 6).

**Figure 7:**
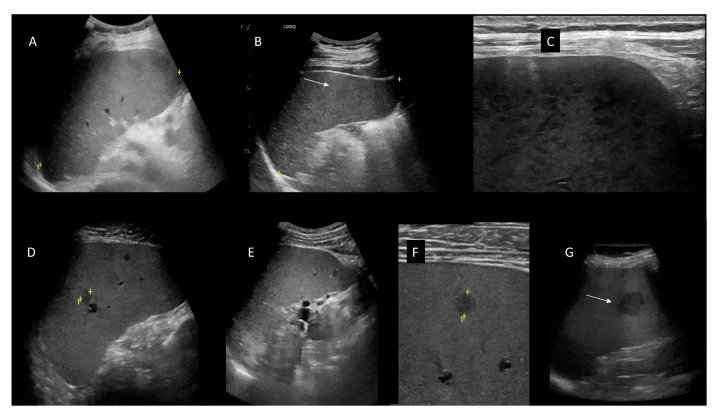
Patterns of splenic involvement in CLL patient. (**A**) Homogeneous splenomegaly. (**B**) Finely inhomogeneous diffuse infiltration of the spleen. (**C**) Micronodular diffuse B infiltration of the spleen (image obtained with a high-frequency linear probe to show the details). (**D**,**E**) Micronodular disperse hypoechoic lesions (white arrows). In panel (**D**) two focal, micronodular hypoechoic focal lesions are found using a convex probe, one meso-splenic and the other at the inferior pole of the spleen; in (**E**) one hypoechoic lesion found using a convex probe is shown. (**F**) The same hypoechoic lesion is shown, by scanning with a high-frequency linear probe. (**G**) Hypoechoic macro-nodular focal CLL lesion of the spleen found using a convex probe (white arrow). In panels “+” signs indicate the position of the calipers to measure the spleen dimensions; “1” indicates the splenic longitudinal diameter.

In N = 22 patients out of 75 CLL patients examined with US, we found multiple hypoechoic areas related to splenic CLL involvement. We found five patterns of infiltration of the spleen due to CLL: (1) homogeneous splenomegaly (N = 7), (2) finely inhomogeneous diffuse infiltration (N = 10), (3) micronodular diffuse (N = 11), (4) micronodular disperse hypoechoic lesions (N = 6), (5) macronodular involvement (N = 5). The pattern of infiltration is undetectable with palpation (Figure 7).

## 4. Discussion

In general practice, according to the 2018 IWCLL criteria, the assessment of superficial pathological LN number and dimensions in patients with CLL either at diagnosis or in follow-up during or post-treatment is based on palpation [1]. The cut-off value of SupLNs to be considered pathological is 10 mm [1,19]. CT assessment of LN is mostly related to dimensional parameters [28] and CT is generally not recommended in general practice because of radiation hazard [1]. Thus, in general practice, palpation of SupLNs is considered the standard of care, and IWCLL guidelines allow the use of US to assess the abdominal visceral involvement due to the disease [1]. The spleen is assessed in general practice either with palpation (Rai and Binet staging systems rely on physical examination and do not require imaging studies) or with US, which is used in some countries [1].

US features of normal and diseased SupLNs and spleen have been previously published [11,13,14,15,16,17,18]. The performances of the last-generation US machines equipped with high-resolution linear probes allow the detection of echo-poor LN down to 3 mm in size and of LN vessels in LN of about 4–5 mm [4]. The results of this study reported in Table 5 confirm these findings [4] because pathological superficial LNs down to 3–4 mm in size were detected by US. US detection of small CLL SupLNs is fundamental in the setting of patients at the onset of the disease, during and post-treatment, when new small diseased CLL LNs, undetectable at palpation, may appear. This allows the clinician to schedule a different US follow-up with closer interval times, to detect the potential progression of the disease, and to assign a targeted therapy. Moreover, US can assess LNs in areas hardly or not reachable by palpation such as supraclavicular and subclavicular regions, respectively [11].

To the best of our knowledge, in this study, we describe for the first time both the US-BMODE and color-doppler characteristics of SupLNs in patients affected by CLL. In the analysis of total N = 1037 superficial LNs we found that the eco-structural, and vascular patterns of diseased CLL SupLNs resemble the characteristics previously described in lymphomas [20,22,27]. Nevertheless, we found that the peculiarity of the pathological echo-vascular structure of SupLNs in CLL is the absence of homogenous pathological patterns, instead of the possible coexistence of all the pathological features described above, which may be present simultaneously in different anatomical regions, or even in the same anatomical region (Figure 3 and Figure 5). This heterogeneously pathological echopattern in CLL patients may be due to the fact that CLL is a systemic disease that may involve contemporaneously the bone marrow, the peripheral blood, and lymphoid organs at various degrees of involvement, at any specific time point of evaluation. Our findings on SupLN echostructure agree with the data previously reported by Ying et al. [22], because we found that in the assessment of SupLNs of CLL patients, one single US parameter is not enough to define the LN as pathological, but all the echo and vascular-structural patterns give an accurate assessment of LNs (Figure 3). In several CLL patients examined in this study indeed, although the L/S ratio of CLL SupLNs was > 2, all the other BMODE and Doppler or microvascular network findings allowed us to define the LN as pathological and diseased by CLL (Figure 3). We also described five different US patterns of involvement of the spleen, which have never been described before in CLL patients. In patients responding to treatment, the spleen returned to normal size, and the echo structure, eventually altered during the disease, showed the recovery of a normal homogenous pattern. This behavior in patients responding to treatment reinforces the finding that not only splenomegaly per se is a sign of splenic involvement by CLL, but also the US patterns described are suggestive of spleen involvement in CLL.

The IWCLL and ESMO guidelines indicate progressive lymphadenopathy or progressive splenomegaly as a sign of active disease [1,19]. It has been previously published that patients with lymphadenopathy with an LN size bigger than 50 mm receiving targeted therapies (BTK of BCL2 inhibitors) have a worse progression-free survival (PFS) compared to those with LNs of smaller dimensions [9,10].

Thus, in the era of new therapeutical options such as BCL2 or BTK inhibitors, a timely definition of progressive disease is important [8,9], and the precise assessment of the number of pathological LNs and mostly of their dimensions is crucial before they reach a dimensional cut-off that is known to be associated with poorer PFS [9,10].

In our study, palpation was considered the standard approach in general practice to assess CLL patients in the outpatient clinic setting, as suggested by the IWCLL and ESMO guidelines [1,19]. Although there was concordance in the detection of the number of superficial pathological LNs between two hematologists (MD1 and MD2) with clinical expertise in lymphoproliferative diseases, the correlation was moderate (*r* = 0.446, *p* < 0.001 and *r* = 0.486, *p* < 0.001 in the inguinal and laterocervical regions, respectively) or absent (*r* = 0.248, *p* = 0.133 in the axillary region) in the description of the dimensions of the pathological LNs. The palpation of the axillary region may be indeed particularly difficult with respect to the laterocervical anatomical region, due to the presence of adipose tissue. This may lead to possible bias in the clinical judgment.

We found concordance between two different US machines from two different US manufacturers, to assess both superficial LNs and splenic dimensions. This suggests that the assessment using US can be reproduced in other hematology units independently by their US machine equipment.

Comparing the results of palpation of SupLNs vs. US, we found discordance in the number and dimensions of pathological LNs in all superficial anatomical regions. US with both sonographers detected a higher number of pathological CLL LNs with respect to palpation. Moreover, this study found that palpation may miss remarkable-sized pathological LNs and splenomegaly (Table 5). Our findings agree with Saiag et al. and Prayer et al. who previously published that US is more precise over palpation to assess metastatic SupLNs [29,30].

We might speculate that the highest discordance in LN dimension assessment between US and palpation in the axillary and inguinal regions, might be due also in this case to the anatomical adipose tissue present in both regions, and, in addition, to the deep anatomical position in the axilla.

Thus, in our study, we found that US could be a valid tool to detect more precisely than palpation the lymph nodal and splenic progression of disease, allowing to find patients who are candidates to initiate treatment or even to change treatment before LNs reach dimensions that impact unfavorably on PFS [9,10].

Moreover, as previously reported [12], US can assess the remission status of patients treated with Venetoclax in clinical practice even though LN dimensions exceed 15 mm, which is the cut-off to define CR [1,20]. US reveals indeed liposclerotic LN that may change a dimensionally-based partial remission into a CR according to the remission observed in the bone marrow and peripheral blood [12]. This observation suggests that in patients undergoing targeted therapies, US is a reliable tool in general practice to avoid possible false-positive signs of nodal progression of disease while patients are still responding to therapy [11]. This implies a crucial decision on the therapy continuation or variation.

Regarding the spleen assessment, US revealed N = 39 patients with splenomegaly, in which palpation found normal-sized spleens. Palpation missed splenomegaly up to a maximum of 18.4 cm in longitudinal diameter and 135 cm^2^ in cross-sectional area [23]. Thus, US is accurate to detect signs of progressive splenic disease [5].

We may hypothesize that this discordance is due to the presence of gas in the bowel, especially in the right colonic flexure, that could mask the correct palpation of the inferior splenic pole. This inconvenience does not apply to US assessment of splenic dimensions because US is not influenced by the gas present in the bowel [5].

## 5. Conclusions

Although currently palpation is considered the standard of care outside clinical trials, our study has demonstrated in a prospective cross-sectional analysis that US allows to find in CLL patients a statistically significant number of pathological SupLNs higher than palpation, that palpation may miss remarkable sized CLL SupLNs, and the real dimensions of involvement of the spleen. Thus, US appears more reliable to detect LN and splenic signs of active and progressive disease. In the era of chemo-free targeted therapies such as BTK and BCL2 inhibitors, non-invasive, radiation-free, low-cost US assessment of SupLNs and of the spleen, in general practice, may be a reliable tool to monitor disease response during treatment, and to detect signs of progressive disease allowing to either re-schedule patients follow up or to shift patients from one treatment to another, in order to reach a deep response up to minimal residual disease (MRD negativity), and to improve PFS. The independence of our results from the US equipment, the BMI, and age, makes this study suitable for a multicenter prospective study.

## Figures and Tables

**Table 1:** Statistical results obtained by the comparison of the total number of pathological SupLN found by MD1 and MD2 by palpation of SupLN. R = right side, L = left side.

Superficial LN Region	Cohen’s Kappa
Inguinal R	0.425
Inguinal L	0.754
Axillary R	0.324
Axillary L	0.663
Laterocervical R	0.649
Laterocarvical L	0.660

**Table 2:** Statistical results of the comparison between MD1 and MD2 related to the assessment of the dimensions in mm of the cervical, axillary, and later cervical SupLNs classified as pathological using palpation. In the last two columns, the correlation analysis (Pearson’s *r*) is also reported. N = number of LN; SD = standard deviation.

		Mean Comparison (mm)			Correlation Analysis	
Superficial LN Region	N	Mean (mm)	SD (mm)	*p*-Value	Pearson’s r	*p*-Value
Inguinal MD1	89	9	7	0.005	0.446	<0.001
Inguinal MD2	89	7	5			
Axillary MD1	38	7	6	0.017	0.248	0.133
Axillary MD2	38	4	4			
Laterocervical MD1	69	8	5	<0.001	0.486	<0.001
Laterocervical MD2	69	5	5			

**Table 3:** Comparison of the dimensions of bilateral SupLNs in the sub- and supraclavicular regions and of the spleen (US1 vs. US2) obtained using two different sonographers (US1 vs. US2). In the last two columns, the correlation analysis (Pearson’s *r*) is also reported. US1 = Esaote ultrasonographer; SD = standard deviation; US2 = GE ultrasonographer; R = right side, L = left side. CSarea = cross-sectional area (cm^2^). Long diam = longitudinal diameter (mm).

	Mean Comparison (mm)				Correlation Analysis	
Superficial LN Dimensions	N	Mean (mm)	SD (mm)	*p*-Value	Pearson’s r	*p*-Value
US1_Subclavicular_R	37	11.03	4.45	0.480	0.992	<0.001
US2_ Subclavicular_R	37	11.19	4.18			
US1_ Subclavicular_L	46	9.47	2.83	0.259	0.993	<0.001
US2_ Subclavicular_L	46	9.30	2.66			
US1_Sovraclavicular_R	14	9.67	2.75	1.000	0.996	<0.001
US2_Sovraclavicular_R	14	9.67	2.58			
US1_Sovraclavicular_L	9	9.03	3.69	0.307	0.994	<0.001
US2_Sovraclavicular_L	9	9.24	3.35			
Spleen dimension		Mean (mm)	SD (mm)	*p*-value	r	*p*-value
US1_splenic_Longdiam	75	13.66	2.79	0.276	0.981	<0.001
US2_splenic_Longdiam	75	13.46	2.83			
US1_splenic_CSarea	75	67.30	31.56	0.381	0.967	<0.001
US2_splenic_CSarea	75	64.47	25.12			

**Table 4:** Comparison between the results of US and palpation assessment describes the number of pathological CLL SupLN and the number of patients with splenomegaly. SupLNs are classified as normal if they are either reactive or liposclerotic with US, or <10 mm with palpation. Spleen is classified as normal if not palpable below the costal margin (by palpation) or if the longitudinal diameter is <12 cm, and/or cross-sectional area is <45 cm^2^ with US.

		Normal	*Pathological*	*Cohen’s Kappa*
		Spleen (Palpation)		
Spleen US	Normal	14	0	0.063
	Pathological	51	10	
		R-Ing_palpation		
R-Ing. US	Normal	19	1	0.031
	Pathological	101	18	
		L-Ing_palpations		
L-Ing. US	Normal	17	3	0.001
	Pathological	100	21	
		R-Ax-palpation		
R-Ax-US	Normal	10	0	0.001
	Path	160	8	
		L-Ax-palpation		
L-Ax-US	Normal	3	0	0.001
	Pathological	168	5	
		R-Lcv-palpation		
R-Lcv-US	Normal	8	1	0.005
	Pathological	149	16	
		L-Lcv-palpation		
L-Lcv-US	Norm	8	0	0.001
	Pathological	170	10	

**Table 5:** Dimensions of SupLNs and spleen detected by US not detected by palpation.

SupLNs	Median (Range) (mm)
Right inguinal	18.6 (5.5–45)
Left inguinal	18 (3.0–43.5)
Right axillary	17 (5–54)
Left axillary	18.6 (6–54)
Right sub-clavicular	10 (4.3–31)
Left sub-clavicular	9 (6.0–20)
Right supraclavicular	9.5 (7.7–30.6)
Left supraclavicular	14 (6–21)
Right laterocervical	13.5 (4–34.5)
Left laterocervical	12.1 (4–33)
Spleen	Median (range)
Longitudinal diameter (cm)	13.8 (12.5–18.4)
Area (in cm^2^)	69 (47–135)

## Data Availability

The datasets generated and analyzed during the current study are available from the corresponding author on reasonable request.
